# Description and phenotype of a novel C5 gene mutation and a novel combination: family report and literature review

**DOI:** 10.3389/fimmu.2025.1605903

**Published:** 2025-07-07

**Authors:** Asier Lizama-Muñoz, Juan Francisco Gutiérrez-Bautista, Monica Bernal, Miguel Ángel López-Nevot

**Affiliations:** ^1^ Department of Biochemistry, Molecular Biology and Immunology III, Faculty of Medicine, University of Granada, Granada, Spain; ^2^ Clinical analysis and Immunology Department, University Hospital Virgen de las Nieves, Granada, Spain; ^3^ Institute for Biosanitary Research of Granada (ibs.GRANADA), Granada, Spain

**Keywords:** complement deficiency, C5 deficiency, C5 mutation, novel mutation, phenotype and genotype

## Abstract

**Background:**

Patients with C5 mutations are more susceptibility to Gram-negative bacterial infections, particularly *Neisseria* species.

**Objective:**

To describe the phenotype and clinical features of a family carrying two C5 gene variants, including one novel mutation, and to assess their functional and genetic significance.

**Methods:**

We analyzed the clinical and genetic characteristics of a family with two compounds heterozygous C5 variants. Clinical features were assessed across affected and unaffected family members, and results were correlated with genetic and functional assays.

**Results:**

Genetic testing revealed compound heterozygous variants in the C5 gene: c.713T>C (p.Ile238Thr) and c.1949G>T (p.Gly650Val). The p.Ile238Thr variant, located in exon 7, results in a substitution of isoleucine with threonine. The p.Gly650Val variant, located in exon 15, replaces glycine with valine. Sanger sequencing confirmed the variants were in trans (on separate alleles). The mother carried the same two variants as the patient. Two siblings carried one variant each (Gly650Val and Ile238Thr, respectively), and one sibling was homozygous for the Ile238Thr variant.

Clinically, the patient, the mother, and the homozygous sibling had very low serum C5 protein and CH50 levels, correlating with increased susceptibility to *Neisseria* infections. Siblings carrying only one variant had normal complement function. *In silico* analysis and molecular modeling indicate that both amino acid substitutions (Ile238Thr and Gly650Val) may disrupt C5 protein structure. The Ile238Thr change introduces a polar residue in place of a hydrophobic one, disrupting the hydrophobic core and opening a loop between beta-sheets. The Gly650Val change substitutes a small residue with a larger one, causing steric hindrance that necessitates structural rearrangements, including shifts in a loop, alpha-helix, and beta-sheet.

**Conclusion:**

We describe a novel C5 variant (Gly650Val) a previously reported variant (Ile238Thr) in unique genotypic combinations (compound heterozygous and homozygous) associated with marked C5 deficiency and increased susceptibility to invasive Neisseria infections. Our findings underscore the importance of combining genetic, functional, and structural data for variant interpretation in complement deficiencies.

## Introduction

The complement system is a fundamental component of innate immunity, playing a central role in host defense by facilitating the elimination of pathogens, clearance of immune complexes, and removal of apoptotic cells. Activation occurs via three distinct pathways—classical, lectin, and alternative—all of which converge at the terminal complement cascade. This common pathway involves components C5 through C9, culminating in the assembly of the membrane attack complex (MAC), which forms transmembrane pores that lyse target cells (1, 2).

**Figure 1:**
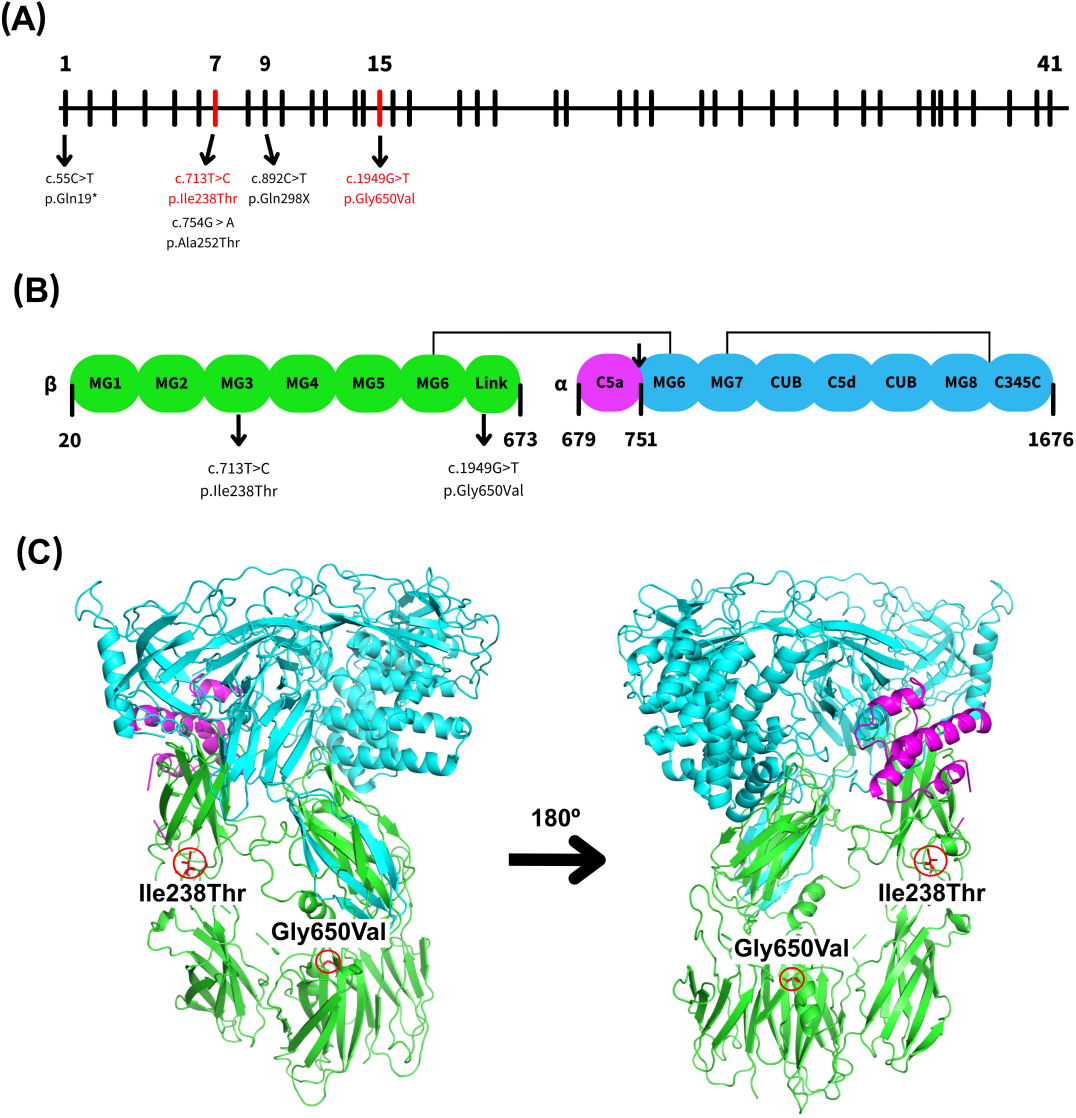
Schematic and structural representation of the human complement component 5 (C5). **(A)** Representation of the genomic structure of the human C5 gene, highlighting exons 7 and 15 in red, which contain the variants identified in this study. Other relevant C5 variants previously reported in nearby exons were also included (3–6). **(B)** Schematic overview of the C5 protein structure, showing the α- and β-chains where the MG1–MG6 domains are shown in green, the C5a domain in purple, and the MG6–MG8 and C345C domains in blue. The cleavage site between C5a and C5b is marked by an arrow, along with selected key residues. There are also indicated domains where the reported variations are ubicated. **(C)** Ribbon diagram of the C5 protein structure generated using PyMOL and PDB entry 3CU7 highlighting the position of both C5 variants identified in this study. Domains are colored as in **(B)**.

The C5 gene, located on chromosome 9q33.2 (3), comprises 41 exons that encode a 1,676-aminoacid precursor protein known as pre-C5 (**Figure 1A**). This precursor contains an arginine-rich linker region (RPRR) spanning residues 674-677, positioned between the N-terminal β-chain and the C-terminal α-chain (**Figure 1B**). Proteolytic cleavage of pre-C5 yields the α-chain (encoded by exons 1–16) and the β-chain (exons 17–41), which remain covalently linked via disulfide bonds (**Figure 1B, C**). C5 is synthesized primarily in the liver but also in monocytes and lymphocytes as an intracellular single-chain precursor (4, 7–9).

Mature human C5 is a 196-kDa plasma glycoprotein composed of two disulfide-linked polypeptide chains: the α-chain (~115 kDa) and the β-chain (~75 kDa) (7, 10). Structurally, C5 contains eight macroglobulin (MG) domains (MG1–MG8), a CUB domain, the C5d and C5a (anaphylatoxin) domains, and an extended linker region located between MG1–MG2 and MG4–MG6 (**Figure 1C**). Both the linker region and C5a are inserted within the MG6 domain. The MG1–MG6 domains adopt a superhelical configuration, while the CUB, C5d, and MG8 domains form a compact superdomain structure (11, 12).

C5 activation is mediated by the C5 convertase, which cleaves the molecule into two fragments: the smaller C5a, a 74-amino-acids peptide derived from the N-terminal region of the α-chain (residues 678–751), and the larger C5b fragment, which initiates the assembly of the MAC (5, 9, 13, 14). The MAC is essential for pathogen lysis, and its formation is impaired if any of the terminal complement components are absent, particularly C5b, which serves as the scaffold for MAC assembly (5, 6). C5a functions as an anaphylatoxin, increasing vascular permeability, inducing smooth muscle contraction, promoting mast cell and basophil degranulation, and recruiting immune cells—especially neutrophils—to the site of infection. In contrast, C5b initiates the lytic phase of the complement cascade by providing the binding site for C6, which subsequently leads to the sequential assembly of C7, C8, and multiple C9 molecules, resulting in the formation of transmembrane pores that disrupt target cell membranes (4, 15).

Complement deficiencies represent a diverse group of inborn errors of immunity and can involve any component of the complement cascade. Among these, C5 deficiency is characterized by absent CH50 and AH50 hemolytic activity, impaired bactericidal function, and increased susceptibility to infections caused by Gram-negative bacteria—particularly *Neisseria* species, which are commonly associated with meningitis and disseminated gonococcal infections (15–18). The most frequently identified genetic alterations in C5 deficiency are nonsense mutations, although many are classified as “variants of uncertain significance” due to a lack of functional or clinical evidence. In this study, we investigated the clinical presentation and genetic background of a family harboring two distinct C5 gene variants—one previously reported and classified as a variant of uncertain significance, and another novel, likely pathogenic variant associated with a clear clinical phenotype.

## Materials and methods

### Patients

We evaluated a patient with a history of multiple episodes of recurrent meningitis caused by Neisseria spp. infections. The clinical presentation raised suspicion of an underlying inborn error of the complement system, prompting comprehensive immunological and genetic evaluation. In addition to the index case, close family members—including the patient’s siblings and mother—were included to investigate inheritance patterns and identify potential genetic variants associated with complement deficiency (**Table 1**). The patient’s father was not included in the study, as he had passed away from colorectal cancer in 2021 at the age of 58. The family was of Caucasian ancestry, and the parents were not consanguineous.

**Table 1:** Demographic data of the family under study.

Family member	Age (years)	Sex	Clinical information
Patient	40	Female	At least two severe meningitis, at the age of 34 and 39
Mother	69	Female	An extremely severe meningitis when she was young
Sibling 1	45	Female	No relevant clinical information
Sibling 2	42	Female	No relevant clinical information
Sibling 3	34	Male	A meningitis at the age of 20

Peripheral blood samples were obtained from all available participants. Total genomic DNA was extracted from peripheral blood mononuclear cells (PBMCs) for genetic analysis, while serum samples were used for functional assessment of the complement system.

This study involving human participants was reviewed and approved by the Portal de Ética de la Investigación Biomédica, Junta de Andalucía (Protocol Code: 0297-N-21). Written informed consent was obtained from all participants before inclusion.

### Molecular analysis

Genomic DNA was analyzed using next-generation sequencing (NGS) with Twist Bioscience kits for molecular amplification and library preparation. Sequencing was performed on the Illumina NextSeq 1000 platform, and data analysis and variant interpretation were conducted using SOPHiA DDM™ for Genomics. Sequence alignment was carried out against the GRCh37/hg19 human reference genome. All identified variants of interest were subsequently validated by targeted Sanger sequencing.

The customized gene panels included the coding regions and flanking intronic sequences of 575 genes associated with primary immunodeficiencies, as previously described (18). These panels also encompassed all complement-related genes, including: *C1QA C1QB*, *C1QC*, *C1QTNF4*, *C1R*, *C1S*, *C2*, *C2orf69*, *C3*, *C4A C4B*, *C5*, *C6*, *C7*, *C8A*, *C8B*, *C8G*, *C9.* The complete gene panel used was included as **Supplementary Materials** (**Supplementary Table 1**).

### Bacterial infection analysis and characterization

The diagnosis of meningitis was established through analysis of cerebrospinal fluid (CSF) and blood cultures. Samples were processed immediately upon receipt by the Department of Microbiology.

Blood cultures were performed using the BD BACTEC FX system (Becton Dickinson), which detected anaerobic bacterial growth within 16 hours. Gram staining revealed the presence of Gram-negative diplococci. Subsequent isolation was carried out on blood agar and chocolate agar, with incubation at 37°C in a 5% CO_2_ atmosphere. After 24 hours, colonies were observed that were smooth, transparent, non-pigmented, and non-hemolytic on blood agar. The oxidase test was positive, and definitive bacterial identification was performed using MALDI-TOF mass spectrometry (MALDI Biotyper, Bruker).

The CSF sample appeared turbid, and Gram-negative diplococci were also observed on Gram stain. Cultures were processed in the same manner as the blood cultures, using both blood agar and chocolate agar, with incubation and identification following the same protocol.

### Gene frequency

Allele frequencies of the identified gene variants were obtained from publicly available population databases, specifically the Genome Aggregation Database (gnomAD) (19) and the Allele Frequency Aggregator (ALFA) database (20).

### C5 and CH50 determination

Serum C5 levels were measured by nephelometry using specific reagents from TRIMERO Diagnostics (Barcelona, Spain) on a BN™ II analyzer (Siemens Healthineers). The method is based on light scattering generated by immune complexes formed between C5 and anti-C5 antibodies. Results were expressed in mg/dL, using a standard calibration curve and internal quality controls provided by the manufacturer.

Total complement activity was assessed using the Autokit CH50 (Wako Chemicals GmbH, Germany), an automated, liposome-based immunoassay that measures activation of the classical complement pathway. The assay relies on the lysis of antibody-coated liposomes containing glucose-6-phosphate dehydrogenase (G6PDH). Upon complement-mediated lysis, G6PDH is released and reacts with NAD and glucose-6-phosphate (G6P), producing NADH, which is quantified by the increase in absorbance at 340 nm. Reactions were conducted at 37°C on an automated analyzer using 10 µL of patient serum 250 µL of reagent R1 (liposomes with G6PDH) and 125 µL reagent R2 (substrate with anti-DNP antibodies, NAD, and G6P). CH50 activity was automatically calculated and expressed in CH50 U/mL using a calibration curve. Internal quality controls were included in each analytical run. Serum samples were either processed immediately or stored at −70°C until analysis. All reagents were stored at 2–10°C according to the manufacturer’s instructions.

### Evaluation of variants pathogenicity

Genomic data was analyzed using the NCBI reference transcript NM_001735.3. The potential impact of the identified variants on protein function was assessed through a comprehensive set of in silico predictive tools, including PolyPhen-2 (21), Mutation T@ster2 (22), Mutation Taster 2021 (23), SIFT (24), LRT (25), MutationsAssessor (26), FATHMM (27), REVEL (28), MetaRNN (29), BayesDel addAF (30). The results of these algorithms are summarized in **Supplementary Tables 2, 3**. Variant interpretation was further guided by the American College of Medical Genetics and Genomics (ACMG) classification criteria (31).

To explore the structural impact of the variants, in silico molecular modeling and structural analysis of the C5 protein were performed. A three-dimensional representation was used to assess how the amino acid substitutions could affect local structural environments and functional domains. PyMOL (version 2.5.7, Schrödinger, LLC) (32) was used for molecular visualization. Structural models of the variants were generated using MODELLER (version 10.6) (33, 34), with default parameters. The native structure of C5 was modeled based on either the crystal structure with PDB identifier 3CU7 (12) or the AlphaFold-predicted structure (AF-P01031-F1) (35, 36). All structural graphics presented in this article were created using PyMOL based on these models (32).

## Results

### Clinical features

The index patient experienced two severe episodes of Neisseria spp. meningitis requiring hospitalization, the first in 2018 at the age of 34, and the second in 2023 at age 39 (**Table 1**). There was no documented history of similar infections during childhood. The initial episode resulted in septic shock secondary to meningococcal infection and bronchopneumonia, necessitating admission to the intensive care unit (ICU) for 20 days. The second episode also presented with clinical features of meningitis and required ICU hospitalization for 14 days. In both instances, the microbiological diagnosis was performed as described in the Materials and Methods section.

Treatment included intravenous antibiotics and dexamethasone for inflammation control when indicated. Following full clinical recovery, the patient was vaccinated against Neisseria meningitidis. Laboratory investigations revealed a marked absence of C5 protein and a severely reduced CH50 activity, consistent with a complement deficiency.

Genetic testing also identified a homozygous variant in the coagulation factor XII gene, associated with increased thrombotic risk. However, the patient was also found to carry the Leu35 variant in factor XIII, which is considered protective against thrombosis, suggesting a low overall thrombotic risk. Additionally, the patient underwent a right hemithyroidectomy and currently reports chronic daily headaches, which are under ongoing clinical evaluation.

Interestingly, the patient’s mother had a documented history of a life-threatening Neisseria infection with sepsis during her youth. One of the patient’s siblings also reported symptoms suggestive of a complement deficiency, including an episode of meningitis at the age of 20 (**Table 1**). However, no further diagnostic evaluations have been performed to confirm this suspicion. No other relevant symptoms or clinical manifestations related to complement deficiency were identified in the family’s medical history.

The index case and her siblings had received vaccination against Neisseria meningitidis during adolescence, following the Spanish national immunization program. The mother had not been vaccinated previously, likely due to age-related exclusion from the program. Following the diagnosis of meningitis in the index case, the patient, her mother, and all siblings received booster immunization, including vaccination against Neisseria meningitidis and the 20-valent pneumococcal conjugate vaccine, as a precautionary measure.

### Genetic analysis

To investigate the underlying cause of the patient’s recurrent *Neisseria* **spp.** infections, molecular analysis of genomic DNA was performed. Two distinct single nucleotide variants (SNVs) in the C5 gene were identified. The first variant, rs567288479 – c.713T>C (p.Ile238Thr), results in the substitution of isoleucine by threonine at residue 238, located in exon 7 within the MG-3 domain (**Figure 1**). This substitution replaces a nonpolar, hydrophobic amino acid with a polar, uncharged residue. The second variant, c.1949G>T (p.Gly650Val), leads to a change from glycine to valine at residue 650, located in exon 15 within the linker region (**Figure 1**). This substitution involves replacing a small, flexible amino acid with a bulkier, hydrophobic one, potentially altering local structural conformation.

The p.Ile238Thr variant has been previously reported by Rosain et al. (2017) and El Sissy et al. (2019) (20, 37), and is listed as a variant of uncertain significance (VUS) in public databases (38, 39). In contrast, the p.Gly650Val variant is novel and, to our knowledge, has not been previously described in the literature or variant databases.

**Figure 2:**
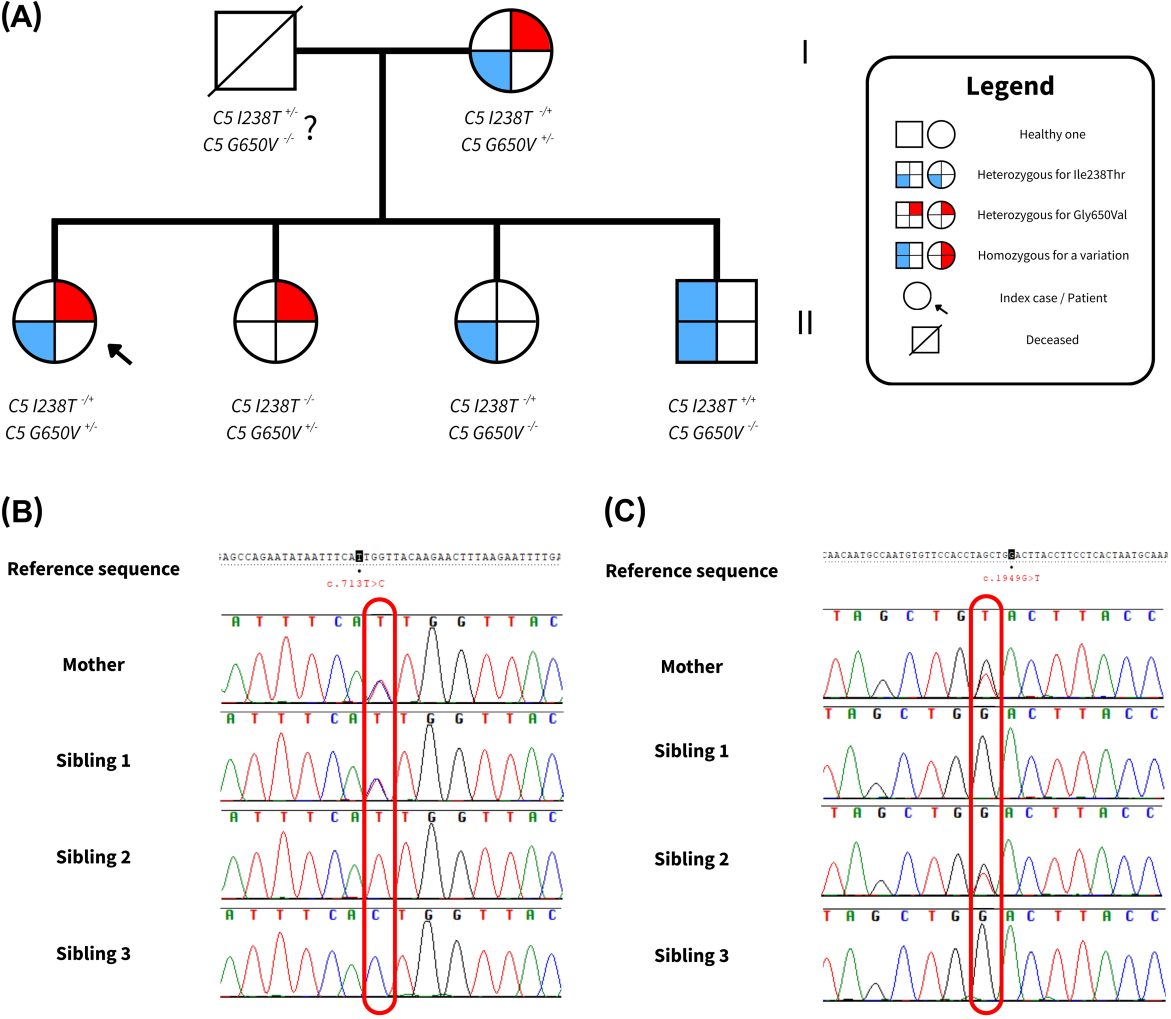
Sequencing outcomes of the family under study. **(A)** Pedigree of the family analyzed in this study. Each figure subdivision represents a C5 allele. The left side of each symbol indicates the presence of the p.Ile238Thr variant, while the right side indicates the p.Gly650Val variant. Shading denotes whether the variant is present in heterozygous or homozygous form, resulting in different allele combinations. **(B)** Sanger results for the c.713T>C (p.Ile238Thr) variant. **(C)** Sanger findings for the c.1949G>T (p.Gly650Val) variant. Complete results from Sanger Sequencing can be found in **Supplementary Figure 1**.

Initially, the phase of the variants—whether in cis (on the same allele) or in trans (on opposite alleles) was unknown. To resolve this, targeted Sanger sequencing was performed in close family members, including the patient’s three siblings and mother (**Table 2**). The results are illustrated in **Figure 2**, and the Sanger chromatograms are provided in the **Supplementary Material** (**Supplementary Figure 1**).

**Table 2:** Genetic analysis results of the genomic DNA sequencing. .

Family member	Locus	Nucleotide variation	Amino acid variation	Carried	ClinVar classification
Patient	Chr 9q33.2 Chr 9:120.93-121.08	c.713T>C;c.1949G>T	p.Ile238Thr; p.Gly650Val	Heterozygous in trans	Uncertain significance; Not described
Mother	Chr 9q33.2 Chr 9:120.93-121.08	c.713T>C;c.1949G>T	p.Ile238Thr; p.Gly650Val	Heterozygous in trans	Uncertain significance; Not described
Sibling 1	Chr 9q33.2 Chr 9:120.93-121.08	c.1949G>T	p.Gly650Val	Heterozygous	Not described
Sibling 2	Chr 9q33.2 Chr 9:120.93-121.08	c.713T>C	p.Ile238Thr	Heterozygous	Uncertain significance
Sibling 3	Chr 9q33.2 Chr 9:120.93-121.08	c.713T>C;c.713T>C	p.Ile238Thr; p.Ile238Thr	Homozygous	Uncertain significance

Chr, chromosome.

Interestingly, the patient’s mother carried both variants, raising the possibility of a cis configuration. However, further analysis showed that two siblings (Siblings 1 and 2) carried only one heterozygous variant each, likely inherited from the mother, while a third sibling (Sibling 3) was homozygous for the p.Ile238Thr variant. This segregation pattern strongly supports a trans configuration in both the mother and the patient, indicating that the p.Gly650Val variant was inherited maternally and the p.Ile238Thr variant paternally.

Although the father’s DNA was not available for direct testing due to his passing, the observed inheritance pattern within the family confirms compound heterozygosity in the patient. These findings are summarized in **Table 2** and graphically represented in **Figure 2**.

### C5 protein levels

To evaluate the functional impact of the identified variants, we performed a functional assays measuring serum levels of complement components, including total hemolytic activity (CH50) (**Table 3**). C5 and CH50 levels were assessed on at least two separate occasions, and the results were consistent across measurements. The index patient, her mother, and Sibling 3—who is homozygous for the p.Ile238Thr variant—exhibited markedly reduced serum C5 concentrations and significantly decreased CH50 activity. In contrast, Siblings 1 and 2, who are heterozygous carriers of a single variant, showed normal C5 levels and CH50 activity, indicating preserved complement function.

**Table 3:** Different complement components serum levels for each subject of the family under study.

Family member	C3 (80–190 mg/dL)	C4 (15–55 mg/dL)	C5 (7–18 mg/dL)	C6 (7.1 - 12.8 mg/dL)	C8 (10.7 - 24.9 mg/dL)	CH50 (23–46 U/mL)
Patient	81.3	19.5	**<0.87 (<LoQ)**	11.3	17.2	**<10 (<LoQ)**
Mother	153	28.7	**<0.87 (<LoQ)**	10.7	26	**<10 (<LoQ)**
Sibling 1	87.5	22.3	13.2	7.28	12.1	33
Sibling 2	108	33.7	11.8	11.3	24.2	**15.7**
Sibling 3	82.9	23.3	**<2.8**	11	18.3	**<10 (<LoQ)**

<LoQ, under the limit of quantification. Bold values indicate results outside the normal range.

All other measured complement components measured across family members were within normal reference ranges. Notably, C5 and CH50 levels in individuals with clinical manifestations were extremely low or below the limit of quantification (LoQ) of the assay used, supporting a functional C5 deficiency in these individuals.

### Evaluation of the pathogenicity

According to multiple bioinformatic prediction tools, both identified variants were predominantly classified as likely pathogenic, based on their predicted impact on protein structure and function due to the amino acid substitutions (**Supplementary Table 1, 2**). However, under the criteria established by the ACMG (29), both variants are currently classified as “variants of uncertain significance” (VUS), as they fulfill only one moderate-level criterion (PM2, absence or rarity in population databases).

We next assessed allele frequencies using data from GnomAD (19, 40, 41) and ALFA (20, 41). For the p.Ile238Thr variant, the reported allele frequency in the general population was 0.005112% and 0.04905% in the European (non-Finnish) population according to GnomAD, with no homozygotes individuals reported (**Table 4**). In the ALFA database, the frequencies were even lower: 0.003% in the general population and 0.004% in Europeans. Based on these data, the p.Ile238Thr variant is considered rare (allele frequency < 0.01%). No allele frequency data was available for the p.Gly650Val variant, as it is not present in either database, supporting its classification as a novel variant. To our knowledge, this is the first documented case of a homozygous p.Ile238Thr variant.

**Table 4:** Mutation frequencies of C5 Ile238Thr variant in all data available on GnomAD and ALFA for the general population (total) and for the European (non-Finnish) and European population respectively, where Spanish population is included.

Variant Information	Total - GnomAD	European (non-Finnish) - GnomAD	Total - ALFA	European - ALFA
Allele Count	79	56	11	1
Allele Number	1545390	1141592	35432	26588
Allele Frequency	5.1e-5	4.9e-5	3e-5	4e-5
Number of homozygotes for the variation	0.00	0.00	0.00	0.00
Number of heterozygotes for the variation	N/A	N/A	5.6e-5	7.5e-5

N/A, not available.

To further assess the structural and functional implications of these mutations, we performed in silico structural modeling using MODELLER, and visualized the resulting models with PyMOL.

**Figure 3:**
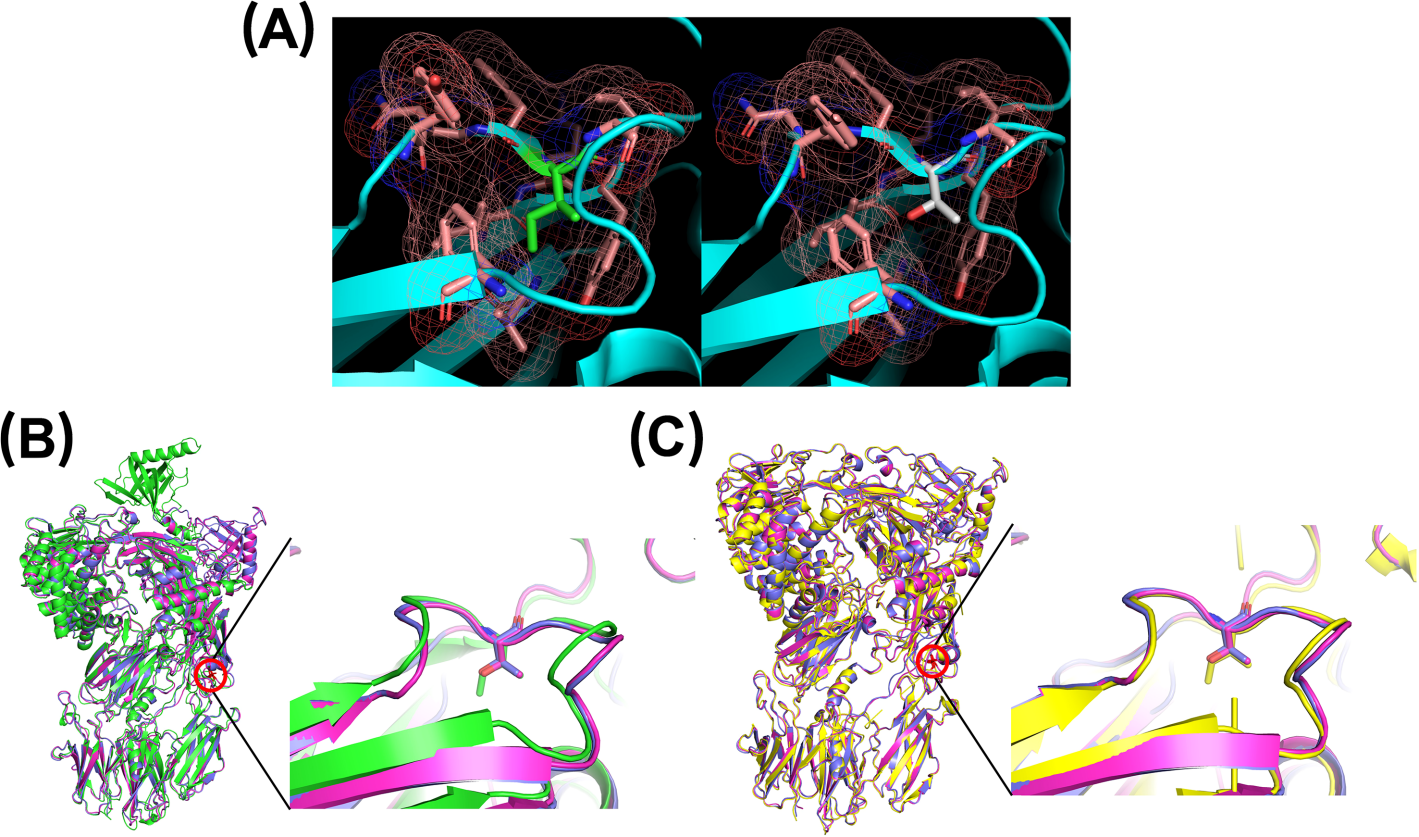
Three-dimensional modeling of residue 238 using PyMOL. **(A)** The native residue, isoleucine (green), and its surrounding environment (pink) are shown on the left. The right panel shows the substitution with threonine (white). **(B)** Comparison of AlphaFold-predicted structure (green) with two MODELLER-generated mutants (magenta and pink). **(C)** Comparison of PDB 3CU7 structure (yellow) with the same two MODELLER-generated mutants (magenta and pink).

For the p.Ile238Thr substitution, the replacement of a nonpolar isoleucine with a polar threonine introduces a hydroxyl group into a hydrophobic region. This disrupts the local environment, as confirmed by comparing the native structure (from PDB 3CU7 and AlphaFold prediction) to our modeled mutant structure. The substitution induces a noticeable structural rearrangement, including the opening of a loop between two antiparallel β-sheets where the residue is located (**Figure 3**). These changes are predicted to impair protein stability, folding, or secretion, consistent with prior findings in structurally similar C5 variants (5, 42).

**Figure 4:**
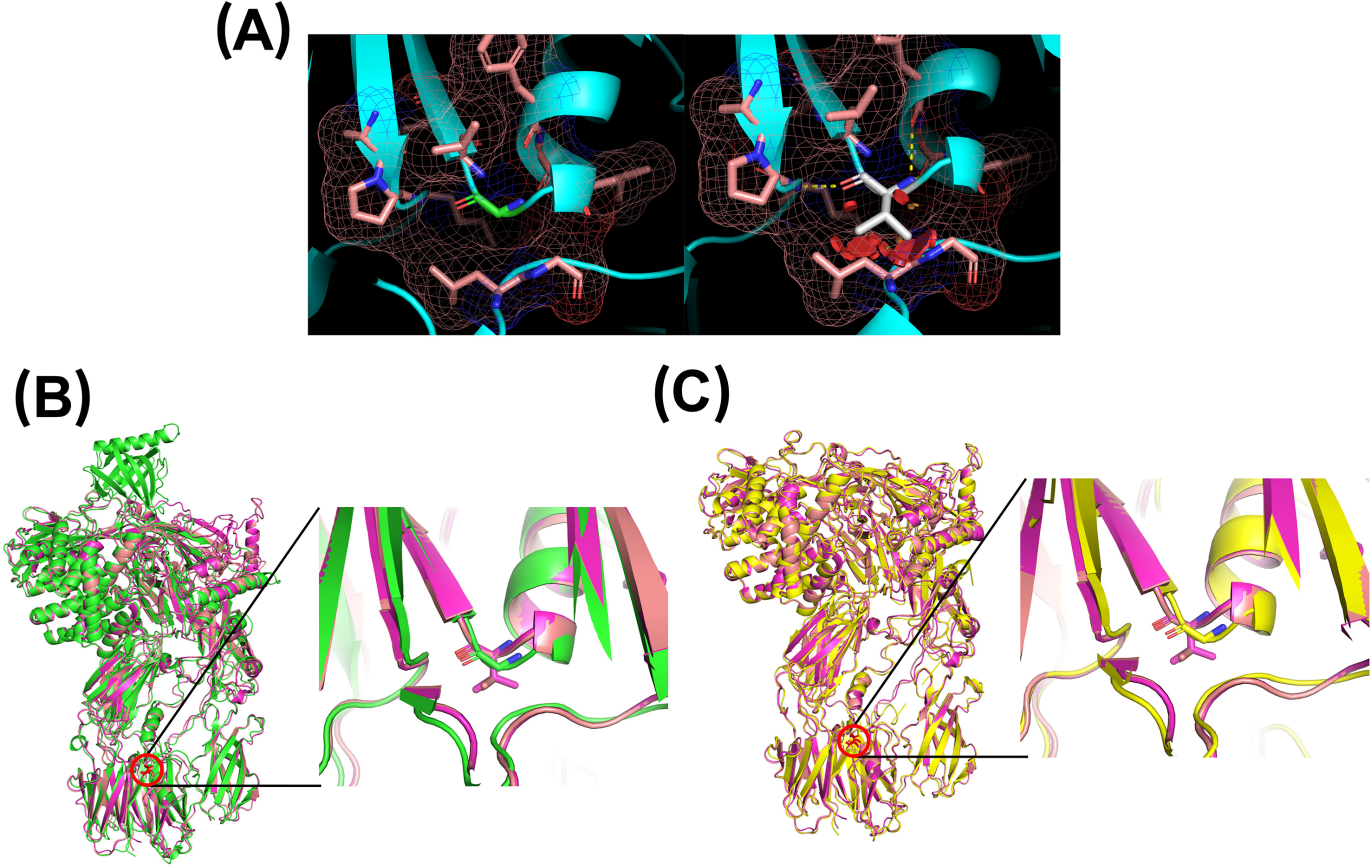
Three-dimensional modeling of residue 650 using PyMOL. **(A)** Left: native glycine residue (green) and its surrounding environment (pink). Right: valine substitution (white), showing potential steric hindrance. **(B)** Comparison of the AlphaFold prediction (green) with two MODELLER-generated mutants (purple and magenta). **(C)** Comparison of PDB 3CU7 structure (yellow) with two MODELLER-generated mutants (purple and magenta).

Similarly, the p.Gly650Val substitution replaces a small, flexible glycine with a bulkier valine residue. Modeling indicated that the valine side chain creates steric clashes that cannot be accommodated without conformational changes to the protein. Comparison of the native and mutant models revealed a clear structural shift, with displacement of the local loop, upward movement of the residue’s position, and adjustment of adjacent secondary structure elements—including a preceding α-helix and a subsequent β-sheet—to minimize steric interference (**Figure 4**). As with the p.Ile238Thr variant, these conformational changes likely compromise the protein stability and function, or interfere with secretion.

## Discussion

Complement C5 deficiency is a rare primary immunodeficiency strongly associated with recurrent bacterial infections, particularly meningitis caused by *Neisseria* spp (18, 43). To date, only a limited number of pathogenic or likely pathogenic mutations in the C5 gene have been reported, accompanied by supporting clinical and functional evidence. In contrast, a significant number of C5 variants are still classified as VUS according to ClinVar (38, 39). In this study, we identified two C5 variants: a novel mutation c.1949G>T (p.Gly650Val), not previously reported, and a previously reported variant c.713T>C (p.Ile238Thr),observed here in the homozygous state for the first time.

Deficiencies in terminal complement components (C5–C9) are known to increased susceptibility to Gram-negative bacterial infections, especially those caused by *Neisseria* spp (16). The formation of the MAC depends on the availability of these components—especially C5b, which initiates MAC assembly (5). Importantly, approximately 50% of normal C5 protein level are sufficient to maintain immune function, and heterozygous carriers of C5 mutations are typically asymptomatic. In fact, cases of asymptomatic C5 deficiency have been reported in the literature (5, 14).

In our study, the index patient, her mother, and a sibling homozygous for p.Ile238Thr exhibited extremely low serum C5 levels and markedly reduced CH50 activity. In contrast, heterozygous carriers of either variant had normal C5 concentrations. Notably, the sibling carrying p.Ile238Thr in heterozygous form showed a mild reduction in CH50 activity, suggesting a partial functional impairment despite normal C5 protein levels. This pattern aligns with prior observations in compound heterozygous or homozygous individuals, in whom complement activity is severely impaired or abolished (14, 42, 43).

Population frequency data confirm that the p.Ile238Thr variant is rare, with gnomAD reporting an allele frequency of 0.04905% in the European (non-Finnish) population, and no homozygotes identified (19, 40, 41). ALFA also reports low frequencies (20, 41). Our case thus represents the first documented instance of homozygosity for this variant. The p.Gly650Val variant, by contrast, is absent from both gnomAD and ALFA databases, supporting its classification as a novel variant.

We hypothesize that both variants negatively impact C5 expression or stability, either through protein misfolding or mRNA degradation, mechanisms previously described in similar variants (5, 14). For p.Ile238Thr, located in the MG-3 domain, the substitution of isoleucine by threonine likely alters a hydrophobic region, disrupting the local structure and potentially exerting a dominant-negative effect (44). The MG-3 domain is known to undergo a 23° rotation during activation, adopting a C3-like conformation, and this conformational flexibility is crucial for interdomain interactions (45).

Thus, even if C5 protein levels remain normal in heterozygotes, CH50 activity may be functionally impaired, as seen in Sibling 2. The homozygous sibling (Sibling 3) exhibited both reduced C5 levels and functional deficiency, consistent with structural destabilization.

The p.Gly650Val variant, located in the linker region, affects a highly conserved segment of the protein (12). The substitution of flexible glycine with bulky valine likely induces steric hindrance, resulting in misfolding and rapid degradation. This would explain the markedly low C5 levels and abolished CH50 activity observed in the compound heterozygous patient.

Bioinformatic predictions for p.Ile238Thr were inconsistent: while some tools classified it as benign, most predicted a damaging effect. In contrast, all tools consistently predicted p.Gly650Val to be deleterious. Despite the current ACMG classification of both variants as VUS (31), our findings—including functional data, structural modeling, and supportive literature—suggest that p.Ile238Thr should be reclassified as “likely pathogenic”, a position previously proposed by Rosain et al. (2017) (32–34, 37). Similarly, given its absence from population databases, consistent bioinformatic predictions, and significant structural and functional impact, we propose that p.Gly650Val also meets criteria for “likely pathogenic” status.

## Conclusion

Based on our findings, we identified and characterized a novel C5 variant, p.Gly650Val, which has not been previously described in the literature, as along with a newly reported genotype combination involving the previously known p.Ile238Thr variant in homozygous form. Our data indicate that the p.Gly650Val in heterozygous is not sufficient to cause disease on its own. However, when present in trans with p.Ile238Thr—it is associated with C5 deficiency. Similarly, the p.Ile238Thr variant alone does not appear pathogenic in heterozygosity, but becomes disease-causing when present either in trans with p.Gly650Val or in a homozygosity.

We observed a strong correlation between the Ile238Thr/Gly650Val and Ile238Thr/Ile238Thr genotypes and severely reduced serum C5 levels, accompanied by markedly diminished CH50 activity. These deficiencies are consistent with an increased susceptibility to Gram-negative infections, particularly those caused by *Neisseria* spp. While further investigation is required to full functional and structural impact of these variants, our study provides valuable preliminary evidence supporting their pathogenic potential.

Despite the relevance of these findings, our study has limitations. First, the analysis is based on a single family, limiting the strength of genotype–phenotype correlations. Future studies, including those involving larger cohorts, will be necessary to validate these associations. Second, we were unable to directly confirm paternal inheritance of the Ile238Thr variant due to the father’s passing. Third, discrepancies among in silico predictive tools highlight the inherent limitations of computational analyses and emphasize the need for experimental validation. Ultimately, further functional studies are warranted to elucidate the precise cellular and molecular effects of these variants. These efforts are already underway as part of our ongoing research.

Despite these challenges, our study represents a significant step forward in understanding C5 deficiency and its role in complement-related immunodeficiencies. By characterizing these previously unreported variants, we contribute novel insights that may inform future diagnostic strategies, variant interpretation, and therapeutic approaches in the field of primary immunodeficiencies.

## Data Availability

The data presented in the study are deposited in the National Center for Biotechnology Information (NCBI) repository, accession number 2975015.
