# Supplementary figures and images for: Description and phenotype of a novel C5 gene mutation and a novel combination: family report and literature review
Source: Front Immunol. 2025 Jul 7;16:1605903. doi: 10.3389/fimmu.2025.1605903 (PMC12277378; doi:10.3389/fimmu.2025.1605903)

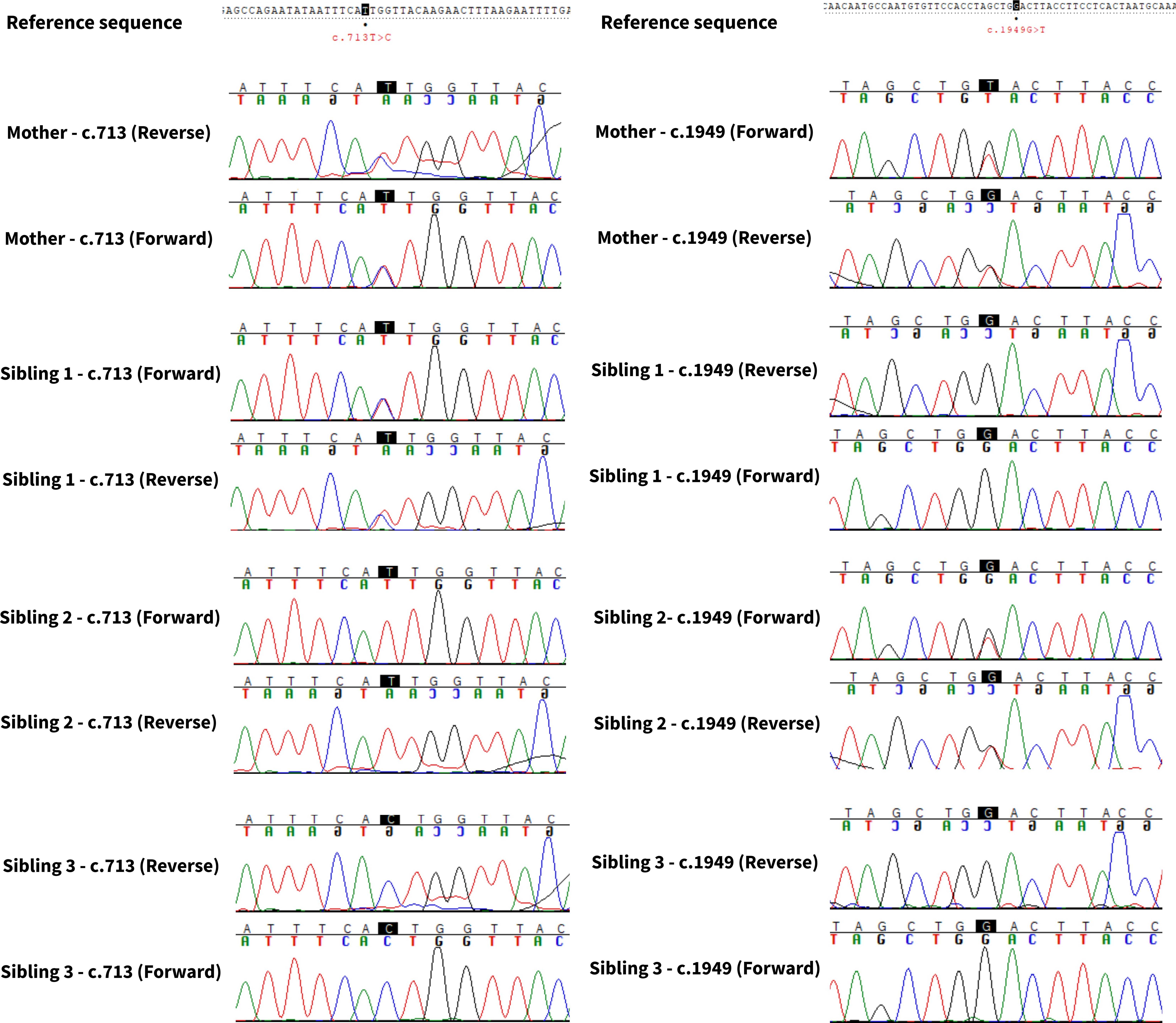

Supplement: Supplementary file 2 [file Image1.jpeg]
